# Less Is More: Latent Learning Is Maximized by Shorter Training Sessions in Auditory Perceptual Learning

**DOI:** 10.1371/journal.pone.0036929

**Published:** 2012-05-14

**Authors:** Katharine Molloy, David R. Moore, Ediz Sohoglu, Sygal Amitay

**Affiliations:** Medical Research Council Institute of Hearing Research, Nottingham, United Kingdom; Ecole Polytechnique Federale de Lausanne, Switzerland

## Abstract

**Background:**

The time course and outcome of perceptual learning can be affected by the length and distribution of practice, but the training regimen parameters that govern these effects have received little systematic study in the auditory domain. We asked whether there was a minimum requirement on the number of trials within a training session for learning to occur, whether there was a maximum limit beyond which additional trials became ineffective, and whether multiple training sessions provided benefit over a single session.

**Methodology/Principal Findings:**

We investigated the efficacy of different regimens that varied in the distribution of practice across training sessions and in the overall amount of practice received on a frequency discrimination task. While learning was relatively robust to variations in regimen, the group with the shortest training sessions (∼8 min) had significantly faster learning in early stages of training than groups with longer sessions. In later stages, the group with the longest training sessions (>1 hr) showed slower learning than the other groups, suggesting overtraining. Between-session improvements were inversely correlated with performance; they were largest at the start of training and reduced as training progressed. In a second experiment we found no additional longer-term improvement in performance, retention, or transfer of learning for a group that trained over 4 sessions (∼4 hr in total) relative to a group that trained for a single session (∼1 hr). However, the mechanisms of learning differed; the single-session group continued to improve in the days following cessation of training, whereas the multi-session group showed no further improvement once training had ceased.

**Conclusions/Significance:**

Shorter training sessions were advantageous because they allowed for more latent, between-session and post-training learning to emerge. These findings suggest that efficient regimens should use short training sessions, and optimized spacing between sessions.

## Introduction

Perceptual learning is the process whereby practice on a perceptual task, such as discriminating between sounds, improves performance on that task. Though learning can be contingent simply on the overall amount of practice [1], it can also be affected by other aspects of the training regimen, including the amount of practice within each session [2], [3], or the length of breaks between sessions ([4] for a review). Systematically investigating the effects of varying the training regimen may provide insight into both learning mechanisms and the optimal design of applied training programs that aim to improve perceptual skills.

When designing training programs, whether for clinical or research use, it is important to use regimens which are feasible for the patient or participant, while ensuring that learning is also maximized. Training sessions that are shorter or fewer in number may increase compliance, especially in children [5]. However, learning may not occur if sessions are too short [1], [2] and may require extensive training, sometimes occurring over thousands of practice trials [6], [7]. Consequently, it is important to find a balance between brevity and efficacy of training. In the experiments described here we addressed two crucial questions: how much training is required overall to produce significant learning, and how is it best distributed across training sessions? In investigating these aspects of learning, the time course of improvements within and across training sessions, and the amount of practice required to trigger and sustain these improvements, are of fundamental importance. In addition, it is necessary to establish the amount of training beyond which no further benefit is gained.

**Figure 1:**
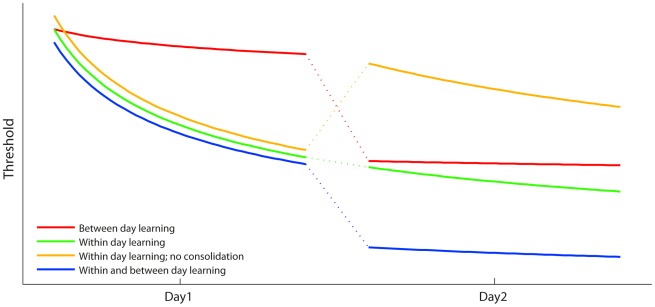
Schema of different time courses for learning. Lines represent different hypothetical learning curves in situations where between- and within-session changes are combined in different ways.

Remarkably different time courses have been observed for perceptual learning. Improvements are often apparent while training (within-session learning [1], [8], [9]; Fig. 1, green line), but can sometimes occur during a latent period after training has finished (between-session learning [2], [7], [10], [11]; Fig. 1, red line). Within- and between-session learning probably represent two different processes, as they can be disrupted independently [12] and show differences in retention [13]. They also appear to have different electrophysiological correlates [13]–[15]. Both types of learning can also occur on the same task [16]–[18] (Fig. 1, blue line). However, neither of two previous studies that varied the number of trials within sessions, while controlling the total amount of practice, reported both forms of learning: Aberg et al. [1] showed only within-session learning in a visual experiment, while Wright & Sabin [2] showed only between-session learning in the auditory domain. Thus, the effect of varying the training regimen on a task that displays both learning types is currently undocumented. Moreover, neither study assessed how well learning was retained once practice had ceased, so the effect of training distribution on long term benefits is unclear.

Perceptual learning studies have shown that specific requirements should be met for learning to occur. For example, a sufficient number of trials (critical minimum) may be required to initiate within-session [1] as well as between-session [2] learning. Insufficient practice results in a lack of performance improvement during training, or a failure of overnight consolidation of improvements attained within a session (Fig. 1, yellow line). On the other hand, learning has been shown with as little as one trial of training on some tasks [3], [19] so minima may not always exist. Within- and between-session learning may have different critical minima, and this can only be established on a task which shows both learning types.

Overtraining on a task is also possible, with extra practice providing no added benefit. For example, no additional between-session learning was observed on a temporal interval discrimination task for a regimen with a large number of trials each day compared with fewer trials [2]. Within-session learning can plateau towards the end of a session and restart once a new session has begun [16], [17], suggesting overtraining within a session. As with the minimum requirements for learning, the maximum effective amount of training may differ for between- and within-session learning, but as of yet no study has determined whether this is the case.

Learning is usually non-linear; very early learning is typically rapid whereas later learning is slower [20], [21]. It is conceivable that other aspects of learning, such as the critical minimum, the maximum effective training per day, or the relative contributions of within- and between-session learning might also change as training progresses. However, studies that have varied the amount of training in each session have used extensive pre-testing to establish baseline performance on the training and untrained tasks, and so the characteristics of the early stages of learning were not documented [1], [2].

Since learning generally follows the characteristic time course described above, large performance improvements occur early on. Extended training may provide only marginal additional benefit compared to less exhaustive training. However, a longer training regimen may provide other benefits: tasks which are learnt over a more extended period of time are often remembered better [4], [22]. On the other hand, longer regimens may produce less generalization than shorter regimens, since as learning progresses it can become more specific to the trained stimulus [6], [23], [24].

Here we used a frequency discrimination (FD) task that was previously shown to result in both within- and between-session learning [8], [25] to investigate the characteristics of early and late within- and between-session learning. We compared their relative contributions to overall improvements, and the parameters within which they produce effective learning. We avoided the extensive pre-tests used in previous studies in order to capture the early stage of learning, and recalled participants up to several weeks after cessation of training to assess retention.

## Experiment 1: Distribution of Training across Sessions

**Figure 2:**
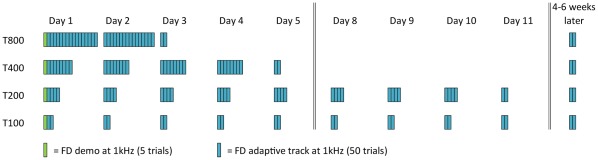
Training regimens for Experiment 1. Groups T800, T400 and T200 trained on 1600 trials of FD overall, with 800, 400 and 200 trials per day, respectively. Group T100 trained on 800 trials of FD overall, with 100 trials per day. A five-trial demo preceded the training, and 100 trials were run the day after training was completed (post-test), and 4–6 weeks after training was completed (retention test).

In this experiment we asked how much training per session is most effective. We varied the number of trials each day whilst keeping the overall amount of training constant (with the exception of the regimen with the shortest sessions). Overall learning and long-term retention were compared between four, multi-day training regimens (Fig. 2). We further assessed whether minimum or maximum effective amounts of daily training were achieved by comparing the speed of learning between regimens. Based on Wright and Sabin [2], who found a critical minimum of between 360 and 900 trials for between-session improvements, we tested a similar range. We expected the group(s) with fewer trials not to achieve a critical minimum and show reduced or no learning. Conversely, we expected overtraining to result in a reduced learning rate in the longer regimens relative to regimens with fewer trials.

We also differentiated learning seen within and between training sessions. We expected to see within-session learning in the early stages of this task, based on previous data from single-session studies in our lab (e.g. [8]). Between-session improvements were predicted to be more dominant in later stages, as typically observed by Wright and colleagues in extended FD training (e.g. [2]).

### Methods

#### Ethics statement

The research protocols for Experiments 1 and 2 were approved by the Nottingham University Hospitals Research Ethics Committee. Informed written consent was obtained from all participants.

#### Participants

Forty eight adults aged 18–27 were recruited via posters from the University of Nottingham student population and the general public, and were paid an inconvenience allowance for their participation. All participants had normal hearing (pure-tone thresholds < = 20 dB HL across 0.5–4 kHz, measured according to BSA guidelines [26]), except one participant who had a threshold of 25 dB HL at 4 kHz in the right ear. Participants had no prior experience of psychoacoustic testing, and had initial FD thresholds between 0.4 and 15% at 1 kHz (i.e. between 4 and 150 Hz), as determined by the first block of trials (see below).

#### General procedure

Participants were allocated to one of the four groups (Fig. 2) according to their FD performance on the first block, in order to match the groups for initial performance. Groups trained using 50-trial blocks of adaptive FD, and differed according to the number of blocks per session. Group T800 trained on 800 trials per day over two days, group T400 trained on 400 trials a day over 4 days, and group T200 trained on 200 trials a day over 8 days (with a weekend occurring after the 5th day) for a total of 1600 trials each. Group T100 trained on 800 trials in total, with 100 trials per day over 8 days (using the same schedule as T200). In all four groups a five trial FD demo was run at the beginning of the experiment to introduce the task concept. FD performance was assessed the day after training was completed (post-test), and four to six weeks later (retention test) using two blocks (100 trials) (Fig. 2).

#### Stimuli

Stimuli consisted of 100 ms tones (including 10 ms raised cosine ramps) presented with an inter-stimulus interval of 500 ms. Stimuli were presented diotically at 60 dB SPL using Sennheiser HD-25-1 headphones. The frequencies of the tones ranged between 1 and 1.5 kHz according to the adaptive procedure described below.

#### Task and adaptive procedure

All testing was conducted within a sound-attenuated booth. The FD task was administered via computer games with a visual interface that cued sound presentation and provided trial-by-trial feedback. Responses were recorded via touchscreen and there was no time limit in which to respond.

During each trial participants heard three intervals, two of which contained a standard tone of frequency f and a third, randomly determined interval, contained a higher-frequency target tone (f + Δf, where Δf is in per cent of the standard frequency f). Participants were instructed to choose the interval that was different from the other two (3-interval, 3-alternative forced choice; 3I-3AFC). The value of Δf was adaptively varied using a three-down one-up staircase procedure, targeting 79.4% correct on the psychometric function [27]. Starting with Δf = 50%, it was divided by 2 following every correct response until the first incorrect response, and then multiplied by two following each incorrect response until the first correct response. Thereafter, Δf was divided by √2 after three correct responses, and multiplied by √2 after one incorrect response. The adaptive track was terminated after 50 trials had elapsed.

A demo of five trials was administered before the first block to familiarize participants with task requirements (see Fig. 2). Three of these trials were ‘easy’ (Δf = 50%), and two were impossible (Δf = 0%). All participants correctly identified the target sounds for the Δf = 50% practice trials.

#### Training, post-test and retention test

Training was administered in blocks of 50 trials of FD, each of which was a threshold assessment where the difference in frequency, Δf, was adapted as described above. Sessions containing more than 200 trials were split up with 5 minute breaks every 200 trials.

All participants completed a further 100 trials of FD (two blocks, identical to those used in training) during the post-test. Some participants (n = 9, 7, 9, 6 for groups T800, T400, T200 and T100 respectively) returned for the retention test, which consisted of a further two blocks of FD.

#### Non-verbal IQ

The matrix reasoning and block design subtests of the Wechsler Abbreviated Scale of Intelligence (WASI [28]) were administered at the end of the post-test to assess non-verbal IQ (NVIQ). A one-way ANOVA confirmed that NVIQ did not differ significantly between the groups (*F*(3,44) = 0.11, *p* = 0.95). NVIQ scores were entered as covariates into all learning ANOVAs.

#### Data analysis

The log-transformed Δf values for each adaptive track were fitted with a logistic psychometric function [29], and the difference limens for frequency (DLFs) were estimated as the 79.4% correct point on this function. Tracks where the psychometric function had a slope of less than 0.10 were discarded because shallow slopes render the threshold estimates unreliable – this occurred for just 0.5% of DLFs measured. One participant was excluded because of highly inconsistent DLFs. Excluding this participant did not affect the mean results, but reduced the variability in the sample considerably.

Overall learning was analyzed by comparing individual DLFs at the beginning of training (average of the first two blocks) and immediately after training (average of the two blocks from the post-test) using a mixed ANCOVA model with group as a between-subjects factor, threshold as the repeated measure, and NVIQ as a covariate. The data were then split to consider the early (first 800 trials) and later (second 800 trials) stages of training. ANCOVAs (as above) were used to compare DLFs at the beginning and immediately after each 800 trials. To compare retention of learning between groups, DLFs at the retention test were modeled using an ANCOVA as described above, but with number of days between post and retention tests as an additional covariate.

To assess whether minimum or maximum amounts of effective training per day had been reached, slopes of the learning curve for each group were compared. Multiple regression models were fitted to the mean DLFs for each block in the first 800 and second 800 trials separately. The models entered log(block) and group*log(block) as covariates, and group was entered as an additional factor for the second 800 trials model to allow for the possibility of different performance levels at the midpoint of training (groups were matched on performance initially). All *p* values of the slope parameters in the regression were Bonferroni corrected for multiple comparisons.

Similarly, to compare the rate of learning over days, mean daily DLFs were modeled using a multiple regression with log(day) and log(day)*group as covariates. Thresholds from block 1 (where all groups were matched on performance) were included in the regression model as the first point. Mean group daily DLFs were calculated by averaging individual thresholds within training days (note that the block 1 DLF was not reused in calculating the mean performance on day 1).

To assess within- and between-session learning, DLFs for the beginning and end of each day were calculated by averaging the DLFs from the first or last two training blocks. As Group T100 only had two blocks per day, it was not included in these statistical analyses. Within- and between-day improvements were calculated for each individual and each day/night, by finding the difference between the relevant DLFs. A multiple regression model with group, log(day) and log(day)*group was fitted to the mean learning for each group (for both within- and between-session datasets), to determine whether the amount of within- and between-session learning changed as training progressed.

### Results

#### Overall learning

**Figure 3:**
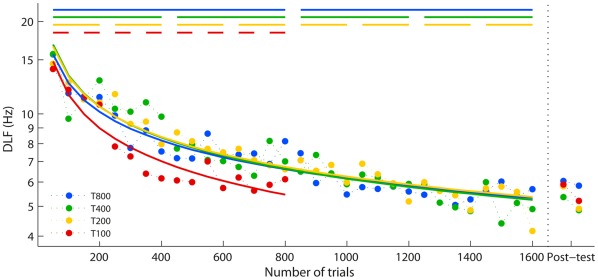
Changes in FD performance with training. Data points show mean group DLFs for each training block of 50 trials, and the post-test. Logarithmic and power curve fits to the mean learning data were compared [43]. Learning was best fitted by a logarithmic function in all groups (power function least squares fits, mean r^2^ = 0.78, logarithmic least squares fits, mean r^2^ = 0.90). Logarithmic fits are indicated by solid curves in the figure. Bars along the top of the figure illustrate sessions in each group’s training regimen. Error bars were omitted for clarity as they overlap for all groups at each block.

DLFs improved significantly (Fig. 3) from the initial blocks to the post-test (*F*(1,42) = 130.2, *p*<.001), with no difference between groups (*F*(3,42) = 0.1, ns). Note that this is true even though the T100 group had half the training. Learning was significant over the first 800 trials in all groups (*F*(1,42) = 92.7, *p*<.001) and also over the second 800 trials in the T200, T400 and T800 groups (*F*(1,31) = 10.3, *p* = .003), with no significant differences between groups at either stage (*F* <0.8 for both analyses).

#### Retention of learning

**Figure 4:**
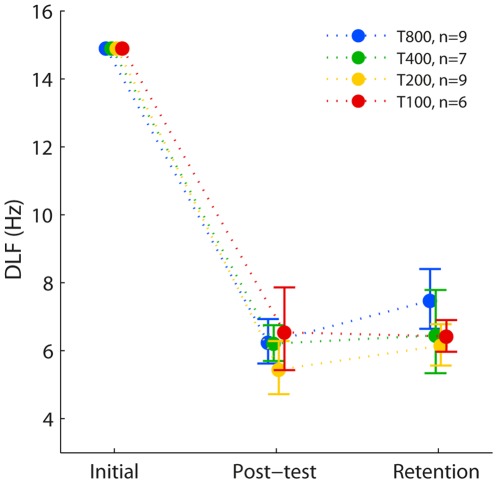
Retention of FD learning following cessation of training. Group mean DLFs for initial performance (first two blocks), post-test (immediately after end of training) and retention test (4–6 weeks later). Groups were no longer matched because only a subset of the participants returned for the retention test, so DLFs were adjusted for individual differences in initial DLFs [44]. Error bars show ±SEM.

Performance did not deteriorate following cessation of training. There was no significant change in DLFs from the post-test to the retention test several weeks later (*F*(1,25) = 0.6, ns), and no difference between groups (*F*(3,25) = 0.6, ns; see Fig. 4), indicating all groups retained their learning equally successfully.

#### Minimum and maximum effective daily training

**Figure 5:**
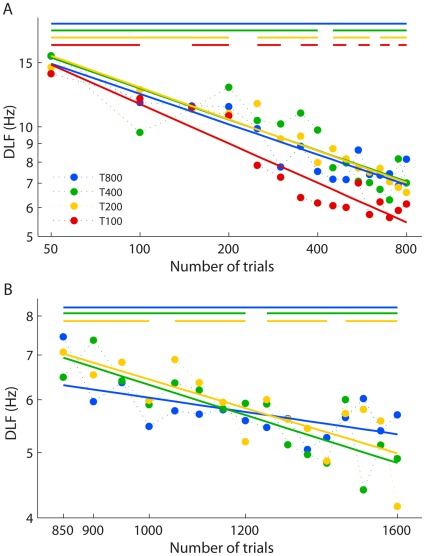
Learning curves for early and late stages of training. (A) Group mean DLFs for the first 800 trials for all groups. (B) Group mean DLFs for the second 800 trials for groups T800, T400 and T200. Solid lines are least squares logarithmic fits plotted on a log-log scale to appear linear. Error bars were omitted, since analyses compared slopes not individual points. Bars along the top of the figure illustrate sessions in each group’s training regimen. Note the different DLF axis scales in A and B.

Learning rates during early (first 800 trials) and later learning (800–1600 trials) were investigated separately (Fig. 5A and B, respectively) by comparing the slopes of the learning curves. During the first 800 training trials the T200, T400 and T800 groups showed equivalent learning speed, but the T100 group showed significantly faster learning than the other groups (*t*(63) >4.9, *p*<.001 for all comparisons). Rather than a critical minimum requirement of daily training, these results suggest that shorter sessions result in more overall learning than longer ones, at least in the early stage of training.

During the second 800 trials (Fig. 5B) the T800 group had a shallower slope than the other two groups, but these differences were not significant after Bonferroni correction (T400: *t*(47) = −2.1, *p* = .042; T200: *t*(47) = −1.9, *p* = .066; α = 0.025). The trend for the T800 group to show slower learning could indicate that 800 trials exceeded a maximum effective amount of daily training in the later stages of learning, with additional trials resulting in less benefit; however further data were required to confirm whether this was the case (see Experiment 2 below).

**Figure 6:**
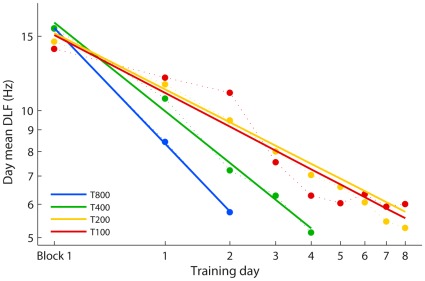
Progress of learning over training days. Group mean DLFs for each training day. DLFs from block 1 are plotted at the far left, followed by daily DLFs for each training day (note that the block 1 DLFs were not reused in calculating the mean for Day 1). Solid lines are least squares logarithmic fits plotted on a log-log scale to appear linear. Error bars were omitted, since analyses compared slopes not individual points.

Considering improvements gained each day rather than per trial further clarifies the relative learning rate (Fig. 6). The slopes describing amount of learning per day grow progressively shallower from T800 to T200, but further reducing the number of trials per day does not decrease the learning rate. The T800 and T400 groups show significant differences in slope compared to each other, the T200 and the T100 groups (*t*(25) >3.7, *p*≤.001). However, the T200 group did not show more daily improvement than the T100 group (*t*(25) = 0.6, ns). This further highlights that the T100 group is improving relatively faster than the other groups, showing as much improvement each day as the T200 group, who had double the training. These results suggest that, at least for this task, 100 trials are above the critical minimum number of trials required to initiate learning, and that there is benefit to having shorter training sessions.

#### Within- and between-session changes

**Figure 7:**
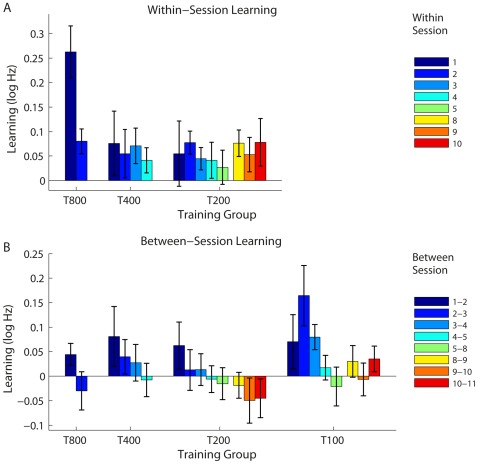
Within- and between-session changes in performance. (A) Group mean within-session changes for groups T800, T400 and T200. (B) Group mean between-session changes in all training groups. The gap between bars 5 and 8 in A and B indicate a weekend break. Error bars show ±SEM.

All groups showed within-session improvements on each day (Fig. 7A). Group T800 showed greater learning on Day 1 than Day 2 (*t*(13)  = −6.8, *p*<.001), but groups T400 and T200 showed no change in the amount learnt in each day (*t*(13) <0.9 for both, ns). The T100 group did not have enough data within each day to be included in this analysis. The results for the T400 and T200 groups suggest that within-session learning is constant, with a fixed benefit per practice block regardless of the stage of training (at least up to 1600 trials). The difference seen in the T800 group’s within-day learning could thus be another indication that while 800 trials per day is an effective regimen for early training, it may lose some efficacy as training progresses.

**Figure 8:**
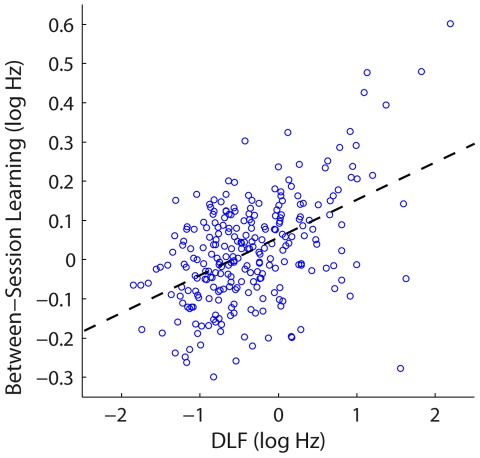
Correlation between performance and between-session learning. Amount of between-session improvement plotted as a function of mean DLFs on the last two blocks of the session. Dashed line indicates the regression fit.

Between-session changes (estimated as the difference in DLFs for the last two blocks of each training day and the first two blocks of the next) were positive at the beginning of training, decreased as training progressed, and became negative in some cases towards the end of training (Fig. 7B). This progressive loss of between session benefit was significant in T800, T400 and T200 (*t*(13) < −5.6, *p*≤.001). T100 data could not be analyzed because there were not enough blocks within each session, but they are pictured in Figure 7B for comparison. Performance at the end of each session (i.e. the average DLF from the last two blocks) was correlated with the between-session change in threshold that followed it – the better the performance, the smaller the between-session gains (r = .49, *p*<.001; Fig. 8).

All four training regimens produced equivalent overall learning and retention. Regimens ranging from as little as 100 trials per day (about 8 minutes’ practice) to 800 trials per day (over one hour of practice) were equally effective on this task, indicating that FD learning is relatively robust to regimen changes. Further, the group with the shortest sessions reached equivalent final performance to that of the groups who trained twice as much overall. This suggests that more training is not necessarily better, and that excess training can, in fact, be inefficient. These findings bode well for applications of FD training, since they support flexibility in the training regimen to suit individual schedules.

Censor and colleagues [30], [31] have also shown improved learning with shorter sessions on a visual texture discrimination task. They attributed their results to within-session adaptation: increased stimulus exposure in longer sessions produced performance deterioration, while overnight sleep resulted in improvement. Our data do not preclude the possibility of adaptation-related deterioration. However, if adaptation did occur, it did not result in reduced within-session learning, as shown by Censor and colleagues. In fact, the greatest within-session learning was observed in the group with the longest training sessions (T800).

The amount of overnight benefit was greatest and lasted over more sessions in the T100 group, consistent with the findings of Goedert and Miller [3] on a visual motor task, where groups trained on fewer trials within a session showed greater overnight improvement than groups who trained on more trials. Our finding that the largest overnight benefit is associated with the poorest performance is also consistent with the observation that difficult tasks (poorer performance) show greater between-session improvement than easier tasks [32]. This is not surprising as performance thresholds decrease with practice. Taken together, these results suggest that using more difficult tasks coupled with short training sessions may be advantageous in maximizing the benefit of between-session learning.

The reduced and negative contribution of between-session learning in later stages of training was unexpected given a previous finding that between-session learning occurs throughout multi-day training, and in spite of extensive pre-tests [2], [33]. It is possible that even extensive pre-testing does not produce much training, and that subsequent between-session learning is still early stage. However, the between-session learning seen by Wright and colleagues persisted over several thousand trials. Here, we saw very little between-session learning after the first 1000 trials. The tones used by Wright and colleagues were very short compared to those used here, making the task more difficult (i.e. increasing the discrimination threshold [34]). Our data show that higher thresholds lead to more between-session learning. Thus, it is possible that harder tasks start with poorer performance and improve more slowly than easier tasks, yielding a later transition from a stage where between-session learning is effective to a stage where it is not. Alternatively, the two tasks may produce different learning profiles because practicing FD on very short tones may train different aspects of auditory perception compared to practice on longer tones.

Differences in the task may also affect the critical minimum number of trials required for learning. Wright and Sabin [2] observed between-session learning for a group who trained on 900 but not 360 trials each day, indicating a critical minimum within this range. The groups in our study (all of whom trained within or below this range) showed no evidence of a critical minimum. It is possible that difficult tasks require more practice within a session in order to trigger learning than easier tasks. Alternatively, as noted above, it may be that fundamentally different aspects of perception are being trained by practice with short and long tones, and that these aspects have different requirements.

While we saw no evidence for a critical minimum, the T800 group showed a marginally reduced slope compared to other regimens in later learning, which could indicate that a maximum amount of effective training was exceeded. One explanation is that within-session learning had saturated during the session, so that some of the practice was wasted. If this were the case, the finding that 800 trials per session was effective for early training would indicate that the effective maximum decreases as learning progresses. This explanation is consistent with the finding that, while within-session learning was constant throughout the study for the T200 and T400 regimens, the T800 group showed less learning within session 2 than session 1. An alternative explanation is that the T800 group (unlike the other groups) did not have any session breaks within the second 800 trials, and so could not benefit from any between-session learning. Our data suggest that this is unlikely; rather than contributing to learning, session breaks produced decrements in performance in these later stages in the other regimens. In addition to investigating the total amount of effective training for lasting performance improvement, Experiment 2 was designed to provide additional data on longer-term training with 800 trials per day.

## Experiment 2: Single- and Multiple-Session Training

**Figure 9:**
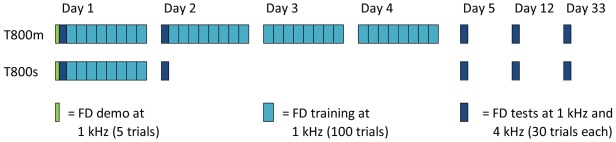
Training regimens for Experiment 2. Two groups trained on 800 trials of FD per day. The T800 m group completed four days of training and the T800 s group completed one. Tests consisted of assessment at the trained and an untrained frequency, and were conducted at the beginning of Days 1, 2 and 5, and then one week (Day 12) and four weeks afterwards (Day 33). A five trial demo preceded the experiment.

The second experiment addressed two questions. The first, raised in the Introduction, is whether extended, multi-day training confers any benefit over single-session training. The second, raised in Experiment 1, regards the possibility that 800 trials per day exceed a maximum effective daily training in the later stages of learning. We found in Experiment 1 that performance of the T800 group improved significantly between 800 and 1600 trials. This would suggest that multi session training should enhance learning compared to single session training. On the other hand, we found that between-session improvements become more negative as training progresses, suggesting prolonged training may be less effective. In Experiment 2 one group trained on 800 trials of FD for a single day (T800 s) and a second group on 800 trials per day over 4 days (T800 m; Fig. 9). All participants were tested at the trained and an untrained frequency before training, and several times during and after training, to determine how well the regimens compared in terms of overall learning, retention of learning, and transfer to another condition.

We expected significant additional learning in the multi-session group compared to the single-session group. Based on visual studies showing increased specificity with training [6], [10], we also expected that multi session training would produce less transfer to a different frequency than single session training. Multi-session learning studies suggest that learning is retained over long time periods [10], [25], [35]. While long-term retention can be observed after extremely short exposure to visual stimuli (for example, the “McCollough Effect” [36], [37]), there is no previous evidence that short auditory training can induce or maintain long-term retention.

If a slower learning rate for T800 regimens in later stages of training is confirmed, data from days 2–4 of the T800 m group will allow us to determine its cause. The slope beyond the first 800 trials should be shallow if a maximum of effective daily training has been exceeded. However, if the slow learning is due to the lack of overnight benefit, the slope over the three additional training days taken together should be equivalent to those of the T400 and T200 regimens in Experiment 1 (Fig. 5B).

### Methods

#### Participants

Thirty adults aged 18–33 were recruited via posters from the Nottingham University student population and the general public, and were paid an inconvenience allowance for their participation. All participants had normal hearing (pure-tone thresholds < = 20 dB HL across 0.5–4 kHz), and had no prior experience of psychoacoustic testing.

#### General procedure

The study consisted of two groups completing one or four days of training, each day comprising 800 trials of FD (Fig. 9). Three tests assessed improvement during training: a pre-test before training began on Day 1, a mid-test at the beginning of Day 2, and a post-test on Day 5. Two further tests assessed retention of learning one week (Day 12) and four weeks (Day 33) after the post-test. A demo of five trials at 1 kHz was run at the beginning of the experiment to introduce the task concept.

The stimuli and task were as described in Experiment 1.

#### Pre-, mid-, post- and retention probe tests

During each test, participants’ DLFs were assessed at both the training frequency f  = 1 kHz and at an untrained frequency f  = 4 kHz. Threshold assessments at each frequency consisted of an adaptive track with 30 trials [38]. The adaptive algorithm was the same as described in Experiment 1.

#### Training

Participants were allocated to either the T800 s or T800 m group based on their pre-test DLFs to match the groups on initial FD ability at 1 kHz. The initial mean group DLFs did not differ between Experiment 1 and Experiment 2 (*F*(1,74) = 0.73, ns). During each session eight training blocks of 100 trials of FD at the training frequency were completed. Blocks consisted of two adaptive tracks of 50 trials, which were interleaved by randomly selecting which of the tracks to use on each trial. Participants were given 10-minute breaks after every four blocks (400 trials).

#### Statistical analysis

DLFs were calculated as described in Experiment 1 for each probe test of 30 trials and each training block (combining the data from both interleaved tracks within the block −100 trials in total). One participant was excluded for the same reasons as in Experiment 1.

Mixed ANOVAs with test day as the repeated measure and group as the between-subjects factor were used to assess learning and retention. Learning was compared from Day 1 to Day 2 to confirm that the groups did not differ during this phase where training was identical. Learning between Day 2 and Day 5 was also tested to see whether the extended training produced any benefit in performance. Retention was assessed by comparing DLFs on Days 5, 12 and 33 between groups, with the Day 2 DLFs as a covariate. These analyses were performed separately for the trained and the untrained frequency. Finally, repeated measures ANOVAs were conducted to compare the total amount of improvement on the trained frequency which occurred after training had ceased (Day 2 for T800 s, Day 5 for T800 m) to the retention test on Day 33.

To check that the training data were equivalent between experiments, a multiple regression was run on the T800 (Experiment 1) and the T800m data from Day 2 (800–1600 trials), with group as a factor, and log(block) and group*log(block) as covariates. To confirm whether there was a slope difference between regimens from 800 to 1600 trials, a multiple regression model was fitted to DLFs from groups T200, T400, T800 (Experiment 1) and T800m, with log(block) as a covariate. A coding variable (H1) was created to test the specific hypothesis that the slopes of groups T800 and T800m differed from those of T200 and T400; H1 was entered as a factor and H1*log(block) was entered as a covariate. Finally the slope of the T800 group on Day 2 was compared to the T800 m slope for Days 2–4, using a regression with group and block entered as described above. Where appropriate, the DLFs from Experiment 1 (based on 50 trials) were averaged across pairs of blocks so that they were comparable to those from Experiment 2 (based on 100 trials).

### Results

#### Learning and retention on the trained frequency

**Figure 10:**
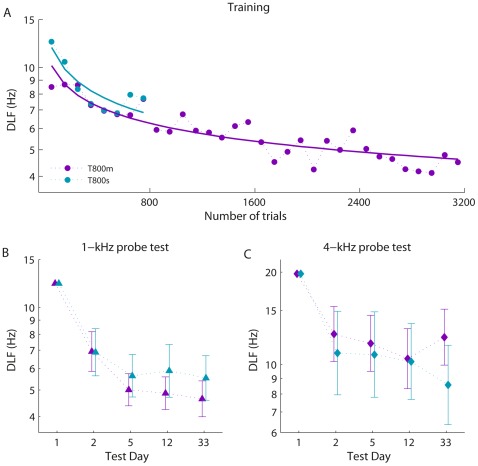
Comparison of single and multi-session training for learning, retention and transfer of learning. (A) Group mean thresholds for training. Solid lines are least squares logarithmic fits. Error bars were omitted for clarity as they overlapped at each point. Note that the pre-test (where groups were initially matched) is not included in this figure or in fitting the learning curves (B) Pre-, post- and retention tests at the trained frequency (1 kHz), adjusted for individual differences in pre-test performance at 1 kHz. (C) Pre-, post- and retention tests at the untrained frequency (4 kHz), adjusted for individual differences in pre-test performance at 4 kHz. Error bars in panels B and C show ±SEM.

Group mean DLFs for the training blocks are shown in Figure 10A, with the tests at the trained frequency in Figure 10B. As expected, both groups showed significant learning between Day 1 and Day 2 (*F*(1,27) = 10.7, *p* = .003), with no difference between them. From Day 2 to Day 5, there was significant additional improvement (*F*(1,27) = 6.2, *p* = .019) but, surprisingly, no significant difference between the T800m group that actively trained during the intervening time and T800 s that did not (*F*(1,27) = 0.4, ns). Performance on Day 5 was maintained by both groups when tested one week and then four weeks later (retention: *F*(2,48) = 0.1, ns; group interaction: *F*(2,48) = 0.3, ns). The T800 s group continued to improve after cessation of training (from Day 2 to Day 33: *F*(1,13) = 17.9, *p* = .001), whereas the T800 m group did not (from Day 5 to Day 33: *F*(1,12) = 0.02, ns). This resulted in the equivalent performance observed on Day 33, suggesting that extended training may be needless since it ‘overrides’ improvements which would occur in its absence, and unlike shorter regimens, may not benefit further from latent learning once training has stopped.

#### Transfer to the untrained frequency

Figure 10C shows performance on the 4 kHz tones before and after training, as well as retention for the same test days as for the trained frequency (see Fig. 9). Both groups showed transfer of learning from 1 kHz to 4 kHz tones following the first 800 trials of training (*F*(1,27) = 11.8, *p* = .002), with no difference between groups. Neither group showed additional improvement from Day 2 to Day 5 (*F*(1,27) = 0.3, ns), and the transfer was retained in both groups from Day 5 to Day 33 (retention: *F*(2,52) = 0.3, ns; group interaction: *F*(2,52) = 1.4, ns).

#### Maximum effective daily training in later learning

**Figure 11:**
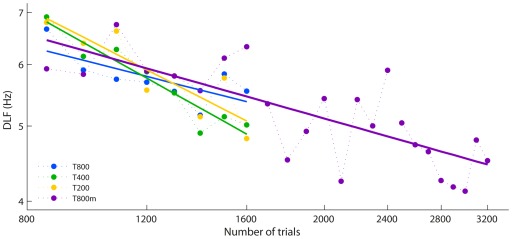
Comparison of learning rates in the later stage of training. Group mean DLFs after the first 800 trials for groups T800, T400 and T200 from Experiment 1 (see Fig. 5B), and the T800 m group from Experiment 2. Data points are mean thresholds for 100 trials each, and solid lines are least squares logarithmic fits. Error bars were omitted, since analyses compared slopes not individual points.

Slopes of the learning curves from 800 to 1600 trials (Fig. 11) did not differ between groups T800 (Experiment 1) and T800m (*t*(15) = 1.7, ns). Combining these data and comparing them to a combination of two groups with fewer daily trials (T400 and T200, which also did not differ, see Fig. 5B) yielded significantly shallower slopes (slower learning) for the groups with 800 trials per day (*t*(31) = 3.8, *p* = .001). This confirms the marginal result from Experiment 1.

The learning slope for group T800 m over Days 2–4 was compared to that of the T800 group on Day 2, and showed no significant difference (*t*(31) = 0.2, ns). This indicates that the reduced learning rate seen for regimens with 800 trials per day is not due to a lack of opportunity for overnight (between-session) gains. Rather, it supports our previous suggestion that 800 trials is above the maximum amount of effective daily practice for later stages of training on this task. This is consistent with the further observation that performance in both groups T800 and T800 m deteriorated towards the end of each session (see Fig. 10A). A trend analysis of block DLFs within sessions showed a significant quadratic term (*F*(1,11) = 10.1, *p* = .009) confirming the upward turn, and this was the case for all sessions (interaction term non-significant).

The single-session group showed significant further improvement after cessation of training whereas the multi-session group did not, resulting in both groups showing equivalent performance on the final retention test (Day 33). Latent performance improvements appear to be greater for tasks which are less well-trained [3], as we have observed above for between-session learning. The latent performance improvements seen after training also extended beyond the overnight benefits found in Experiment 1, with improvements continuing even after the initial between-session benefit between Day 1 and Day 2. This supports findings that improvements can continue for several days after practice has ceased [15].

Our results show that the extended training regimen is redundant for our task. Since performance generally continues to improve as training progresses, it is often assumed that training on fewer sessions would produce less learning in the longer term. One study which did directly compare training on different numbers of days also found that a greater number of training sessions did not improve performance on the trained task compared to fewer sessions [39]. However, we cannot assume that this result will apply broadly. Tasks which produce less daily learning (such as fewer trials each day or a different task in which learning is slower) might continue to show latent performance improvement after more sessions than was seen here. In these situations, multi-session regimens may be beneficial.

We did not observe a difference in the amount of transfer to an untrained frequency between regimens. If specificity increases with training, as suggested by visual studies [6], [23], then 4 days of training on this task is not sufficient for that to occur. However, the transfer of training may not be strictly comparable between auditory and visual learning. According to reverse hierarchy theory (RHT [23], [24]), learning initially occurs in higher areas of neural processing, where receptive fields are large and neurons respond to multiple dimensions of the stimulus. Thus, early learning occurs in areas which are involved in processing a range of stimuli, and will transfer broadly. RHT posits that as training progresses it cascades to lower neural levels, and thus becomes more specific to the trained stimulus. While RHT has received much of its support from visual learning studies, it has been suggested that, due to differences in neural functional architecture between the auditory and visual systems, it does not account for all the processes occurring during auditory learning [40]. Instead, Amitay suggests that, along with learning which proceeds in a top down fashion, in audition some bottom-up learning, associated with improved filtering of irrelevant information, occurs simultaneously. This scheme would imply that specificity would not necessarily increase with training, consistent with results observed here (see also [39]).

Since a no-training control condition was not included in this experiment, we cannot rule out the possibility that the improvement at 4 kHz (which we assume to be transfer from the 1 kHz training) was simply due to repeated testing, a ‘test-retest’ effect. However, if this was perceptual learning of the 4 kHz stimulus induced only by the pre-test assessment, we would have expected learning to continue following the repeated tests (Days 2, 5, etc.). Since such improvement did not continue, we suggest it is transfer associated with the early stage of training on the 1 kHz stimulus, though we cannot entirely rule out it is some form of procedural learning or transfer thereof.

Finally, our results confirmed that 800 trials per day exceeded an effective maximum of training within a session in the later stages of learning. Performance deterioration towards the end of each session for both groups in Experiment 2 suggests that the maximum may be (at least in part) due to fatigue. If within-session learning was simply saturated we would expect performance to plateau, not deteriorate. This deterioration was not observed in the first 800 trials of the T800 group in Experiment 1. In Experiment 2, we suggest it was due to the longer blocks (100 instead of 50 trials) and fewer breaks (every 400 instead of 200 trials), resulting in greater fatigue. Alternatively, it could be indicative of stimulus adaptation, as observed in visual learning [30], [31]. Taken together with the finding that the T800 learning slope for the first 800 trials is as steep as the T400 and T200 regimens, our results suggest that these effects become more prominent as learning progresses.

## Discussion

The most important observation in this study was that the most efficient regimens were those which take advantage of latent learning following training. We observed this learning as between-session improvement in Experiment 1 and as performance gains over several days following cessation of training in Experiment 2. Both results show that the improvements seen after practice has ceased are largest in the early stages of learning. These findings are consistent with previous reports from visual training [3], [32], which indicate dependence of latent learning on the amount of training and the task difficulty. However, our data particularly highlight the role of performance level.

The differences we saw when comparing early and later stages of training reinforce the general observation that learning is not uniform throughout training. While ‘early’ and ‘late’ are clearly not distinct stages – changes occurred gradually – our results highlight the fact that the stage of training is an important factor affecting learning. Between-session or latent learning appears to be most prominent in the early stage of the learning process. Not only should this be taken into account when planning training regimens, but it is also an important consideration when comparing results of different experiments.

The amount of pre-testing used to establish baseline performance varies considerably between studies. Learning can occur during these tests and the subsequent training data reflect different stages of learning, depending on the length and content of the pre-test. However, we have found that even if we take that into consideration, there remain differences between the patterns of subsequent learning reported here and in some other studies. In particular, between-session learning has been found to persist for much longer than would be predicted from our results [2], [33]. We suggest that performance level could be a key factor here; if the more challenging tasks used by Wright and colleagues resulted in performance that was initially worse and improved more slowly, between-session learning may have remained positive for longer. This could explain why participants can train for tens of thousands of trials on some psychoacoustic tasks and still show improvements [6], [7]. It implies that regimens requiring long-term training should endeavor to use tasks which are very difficult, so that between-session improvements remain positive for as long as possible. Indeed, given that it has been shown that increasing difficulty does not prevent learning, even in the limiting case where the task is impossible [8], our results would suggest that training on difficult tasks would always be beneficial.

A further prediction of our findings is that optimal training regimens should have short sessions which are spaced by several days in early learning. This would provide a greater opportunity for between-session, latent improvements while the learning is still positive, as shown in Experiment 1. Longer gaps between sessions would allow between-session learning to reach a maximum over several days, as shown in Experiment 2. Distributed practice (i.e. large gaps between sessions) has previously been found to be more beneficial than massed (consecutive) practice (for reviews see [4], [22]). Our results suggest that the benefit of distributed practice is due to increased opportunity for between-session learning, but as we have noted, this is not seen for all tasks. For example, Aberg and colleagues observed no between-session learning, nor any differences between massed and distributed training [1].

Our results suggest that within- and between-session learning are separable elements of the learning process that develop differently as training progresses. This complements previous findings suggesting that within- and between- session changes are independent: they can be disrupted separately [12], they show differences in retention [13], and they have different electrophysiological correlates [13]–[15]. However, some visual research has suggested a dependence between the two types of learning, with between-session learning occurring only if within-session learning has saturated [16], [17]. Our results do not support any contingency of the two since we observed between-session learning at the beginning of training, when there was no saturation of within-session learning.

The relative contributions of within- and between-session learning change over the course of training. Their interaction is additive in the early stage (when both are positive) and detrimental in the later stage (when between-session learning is negative), suggesting that the classic fast-then-slow shape of the learning curve [20], [21] is driven largely by the changes in between-session learning. This explanation clearly cannot apply to all learning, since fast-then-slow learning curves are found even in experiments where there is no between-session learning [9], [41]. However, it does reinforce the notion that within- and between-session learning can represent independent learning processes, and suggests that modeling of learning curves should consider whether they are better represented separately or by fitting a single curve to overall learning (see [19]).

An important question raised by this study is whether manipulating task difficulty by, for example, using tones of various durations would affect the magnitude of between-session learning. Note that we use ‘difficulty’ here in a constrained sense to refer to tasks for which perceptual thresholds were higher. Other factors such as perceptual load [42] or required cognitive resources could affect difficulty without necessarily impacting perceptual thresholds. If task difficulty in any generalizable sense was found to reliably affect between-session improvements, it could provide a tractable way to enhance learning, which would be of great practical benefit. Additionally, although our results so far suggest that shorter sessions are more efficient, we do not know whether even shorter sessions (<100 trials) would show the enhanced benefit or fail to meet the minimum requirements for learning on this task. The critical minimum may also be affected by the task difficulty – it seems plausible that more difficult tasks would require more practice to initiate learning. Thus, it is important that studies which consider the effect of task difficulty on between-session learning also assess the range of trials per day for which each regimen is effective.

Asking trainees, especially children and older or infirm people, to engage in long periods of sustained engagement with a task is arguably the greatest challenge facing application of perceptual learning. Our results suggest that training is most effective when the opportunity for latent improvement between practice sessions is maximized during early learning. These findings suggest that less intense regimens can be engineered to produce more efficient learning.
